# Longevity: Lesson from Model Organisms

**DOI:** 10.3390/genes10070518

**Published:** 2019-07-09

**Authors:** Giusi Taormina, Federica Ferrante, Salvatore Vieni, Nello Grassi, Antonio Russo, Mario G. Mirisola

**Affiliations:** Dipartimento di Discipline Chirurgiche, Oncologiche e Stomatologiche, Università di Palermo, Via del Vespro 129, 90100 Palermo, Italy

**Keywords:** model systems, aging, signal transduction, molecular senescence

## Abstract

Research on longevity and healthy aging promises to increase our lifespan and decrease the burden of degenerative diseases with important social and economic effects. Many aging theories have been proposed, and important aging pathways have been discovered. Model organisms have had a crucial role in this process because of their short lifespan, cheap maintenance, and manipulation possibilities. Yeasts, worms, fruit flies, or mammalian models such as mice, monkeys, and recently, dogs, have helped shed light on aging processes. Genes and molecular mechanisms that were found to be critical in simple eukaryotic cells and species have been confirmed in humans mainly by the functional analysis of mammalian orthologues. Here, we review conserved aging mechanisms discovered in different model systems that are implicated in human longevity as well and that could be the target of anti-aging interventions in human.

## 1. Introduction

Aging is considered a natural and unavoidable “side effect” of life in spite of the observation that life span can vary greatly between species and individuals. Researchers have developed many theories on the cause of aging, but none of them prevailed. George Williams, an undisputed leader of the field, proposed that natural selection fixes alleles in a population for their positive contribution to fitness early in life, but that the selected alleles become deleterious later in life [1]. Around this idea, he developed the antagonistic pleiotropy theory. According to this proposal, aging is the result of (a) casual selection of late-acting deleterious alleles because of their advantage early in life and (b) the incapacity of natural selection to filter the late-acting detrimental effect of these alleles. Corollary to this theory is that mutations that are capable of increasing the life span must cause a disadvantage or a reduced fitness during the early stages of life. Many mutations that are capable of increasing the life span of different model systems confirmed this prediction, but the existence of long-lived mutants, such as the yeast Ras2 or the *C. elegans* daf-2 that grow and reproduce at a normal rate [2], suggests that extended life doesn’t imply a reproductive or growth rate fitness cost. However, these observation are made in laboratory conditions; therefore, we cannot exclude that these mutants could also show a reduced fitness, but only in the wild.

The disposable soma theory proposes aging as a stochastic process driven by the progressive accumulation of molecular damages within somatic cell lines. This damaging force can be counterbalanced by repair systems that tend to maintain the cellular status quo at some energetic cost. Therefore, the aging rate, which profoundly differs between species, is inversely associated with the amount of energy used for somatic maintenance. According to this theory, periods of nutrients shortage forced organisms to balance the energy used for germ line maintenance as well as reproduction and somatic maintenance [3]. Programmed longevity theory has been proposed as an alternative [4]. It proposes that the healthy portion of life span is programmed to increase the fitness. The aging rate wouldn’t be regulated by an energy-based trade-off between reproduction and longevity, but each species reaches an evolutionary stable strategy. According to this theory, most longevity-extending mutations will cause a trade-off, but some won’t.

However, there is the possibility that the group instead of the individual is the object of evolution. According to this theory, fitness is affected by either “group competition”, “individual competition”, or both, depending on the conditions encountered. The advantage of the single individual is positively selected only if it also confers a group advantage. Mathematical simulations and experimental evidences suggest that at least in *S. cerevisiae*, aging under certain conditions can be programmed, and an altruistic life span extension of the individual can provoke the extinction of the group [4].

None of these theories prevailed so far, which was probably because the complex forces of evolution cannot be simplified by a single scheme. Evolution may be the result of multi-level selection where individual fitness or group fitness are preferred, depending on ecological niche and population density.

**Figure 1:**
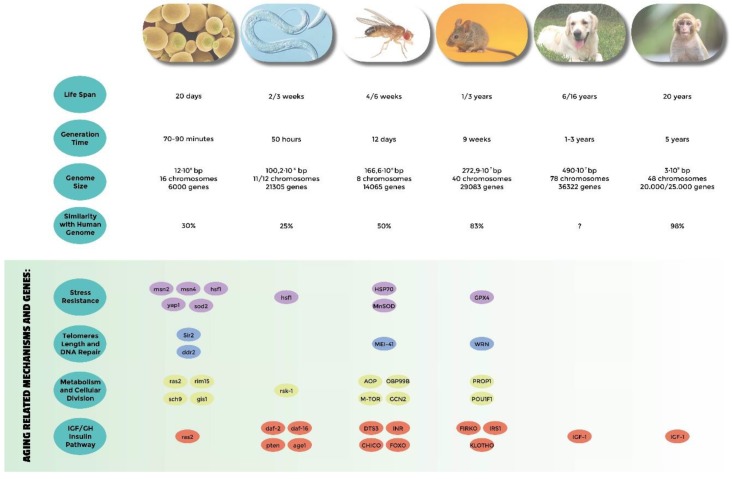
Comparative analysis of the most used model systems in aging research. Genome informations are from NCBI.

Model systems have been widely used to confirm these theories. Their advantage relies on reduced costs, easy maintenance in a laboratory facility, and affordable but rigorous manipulation tools. In general, the simpler the organism we use as a model, the greater these advantages. The short life span of simple organisms is another key advantage of model systems in aging research (Figure 1), which allows both parallel and serial experiments to be performed in reasonable time. In addition, rigorous genetic experiments can be performed in simple eukaryotes using the powerful possibilities of modern molecular genetics, which permit simultaneously screening multiple congenic, or even in some models such as yeasts or isogenic individuals at relatively low cost. The other side of the scale is that some of the simpler systems cannot model alone all the aging phenotypes (e.g., immunosenescence can’t be modeled by yeasts). It is not surprising that, as in many other science fields, biogerontologists thus widely take advantage from the usage of multiple model organisms to explore their theories. In addition, ethical issues are of course much more limited in simple model organisms making *Saccharomyces cerevisiae*, *Caenorhabditis elegans*, *Drosophila melanogaster*, and *Mus musculus* the most popular model systems used in aging research. *Canis lupus familiaris* is becoming an interesting alternative to those model systems because, even though it has a similar ethical constraint to that of humans, massive breed selection offer interesting genetic opportunities. We will describe below the genes and pathways discovered thanks to these organisms that have shed light on possible aging mechanisms in humans. 

## 2. The Simplest Eukaryotic Model: Yeast Cells

Yeast has played a critical role in revealing the molecular genetics of many basic cellular processes such as the cell cycle [5], protein folding [6], intracellular trafficking [7,8], and many others. A short generation time, easy and cheap lab setups, powerful genetic approaches, and high-throughput methodologies [9] are some of the attractive factors of this simple unicellular eukaryote. Genome similarities (homologue and orthologue genes) between this very simple organism and mammalians or even humans are surprisingly high, suggesting that this organism can be an effective model of human diseases [10,11,12]. Two different paradigms of aging exist in yeast: replicative life span (RLS) and chronological life span (CLS) [13]. The first one measures the replicative potential of individual cells. Single cells can in fact be monitored for their ability to generate new cells. Almost 60 years ago, Robert K. Mortimer used this characteristic to count the number of cell divisions obtained from a single cell [14]. In this protocol, the emerging bud (daughter cell) is carefully removed by micromanipulation as it appears until the mother cell stops dividing. The second paradigm (CLS) measures instead the survival of a cellular population in the post-mitotic, non-dividing phase [15]. Thus, the yeast RLS resembles the Hayflick limit observed in cultured mammalian cells, measuring essentially the doubling ability of cultured cells, while the CLS models better the ability to survive and retain the doubling potential of post-mitotic tissues. It is interesting to note that in spite of the clear differences of the two paradigms used to summarize aging in yeast, some pathways such as Ras-PKA or Tor-Sch9 have a consistent role in CLS and RLS [13,16], while others do not (e.g., Sir2 [17]). The yeast life span is doubled through deletion of the gene encoding the small G-protein Ras2, showing a contemporary higher resistance to multiple stresses [18,19]. Ras proteins are molecular switches that can cycle between the on–off state, which correspond to the GTP-bound and GDP-bound, respectively [20]. Mutations on the human orthologue of the yeast Ras are found in 35% of human cancers. The amount of the Ras on-state molecules is dependent on the availability of sugars [21]. Two different pathways are known to be dependent on Ras2 activation in yeast: protein kinase A (PKA) and Mitogen-activated protein kinase (MAPK). Many data suggest a major role of the PKA pathway as a longevity regulator [22]. Msn2/Msn4, two key stress resistance transcription factors, are in fact inhibited by kinase A activity, and Msn2/4 deletion reverts the phenotype observed with Ras2 deletion. In addition, mutations affecting adenylate cyclase, an activator of the kinase A pathway, also increased the survival of yeast cells, confirming the role of the PKA pathway as a major Ras-dependent pathway relevant to the aging processes. PKA activation leads to Rim15 inhibition which, in turn, positively regulates the transcription factors Gis1 [23], Msn2, and Msn4, whose function is to bind respectively the PDS (post-diauxic shift) and STRE (STress Responsive Element) sequences activating the expression of genes involved in survival and stress response such as heat shock proteins, the cytoplasmic catalase, and the two superoxide dismutase (SOD1, SOD2) [24,25], or in DNA repair such as the DDR2 gene [26].

A selection of stress-resistant mutants obtained by transposon mutagenesis was evaluated by chronological life span to isolate long-lived mutants. In this study, the Sch9-Tor pathway emerged as another pro-aging pathway [27,28]. Sch9 is a serine/threonine protein kinase that is homologous to mammalian Akt and S6K. It has been isolated in yeast as a multicopy suppressor of Ras/PKA through the conditional inactivation of the thermosensitive Ras exchange factor allele CDC25ts [29,30]. In addition, the increased G1 length of yeast cells bearing a Sch9 null allele is suppressed by a hyperactive kinase A pathway. These and other data suggest that its function is partly overlapping with the Ras/PKA pathway. The down-regulation of both signaling pathways obtained by specific nutrients depletion results in the greater expression of genes involved in resistance to stresses [31]. The observation that SOD2 expression levels doubles in ras2Δ strains suggests that oxidative stress is a major component of this response [32]. In addition, overactivation of the transcription factors that respond to thermal (HSF1) and oxidative stresses (YAP1) increases survival [33,34]. In spite of these observations, the over-expression of superoxide dismutases and catalases alone does not allow the survival levels obtained by the deletion of RAS2 and SCH9, suggesting that modulation of oxidative stress is not the only mechanism involved in longevity regulation. In addition, despite the aforementioned functional overlap between the Ras and Sch9 pathways, the simultaneous deletion of RAS2 and SCH9 has greater effect than their respective individual deletions. It is important to notice once again that the mediators that are part of these pro-aging cell pathways find their functional or structural orthologues in higher eukaryotes, and it has been confirmed that the mechanisms described are conserved from yeast to mammals. In addition, it has been observed that in all organisms, these pathways are influenced by nutrients, either directly or through insulin/insulin-like growth factor-1 (IGF-1) and growth hormone (GH) in multicellular eukaryotes [35,36,37].

## 3. *Caenorabtidis elegans*

*Caenorhabditis elegans* has been established as a model organism in 1965 by Sidney Brenner. This small size (1.5-mm long adult) soil nematode has great potential for genetic analysis, is easy and cheap at laboratory cultivation, and has a short life cycle: the generation time is only three to five days, and the life span is two to three weeks. *C. elegans* can inbreed by self-fertilization (hermaphrodite). Alternatively, hermaphrodites can cross with males (a possibility that is otherwise known only in plant genetics where selfing and crossing can be manipulated at will). Other key features are the anatomical simplicity (<1000 cells) and the small genome. The latter is about 101-Mbp long, consists of six chromosomes, and contains 19,000 genes, of which 50% are conserved in the human genome. The animal body is transparent, which is a characteristic that allows the tracking of cells in vivo over time, and the fluorescent visualization of tagged proteins. It can be cultivated on agar plates or in liquid media; therefore it is a good system for a wide variety of high-throughput manipulations. In addition, the researchers take advantage of its powerful and continuously expanding genetic and imaging possibilities [38,39]. First, a multicellular organism is completely sequenced, revealing a conservation of ~80% of its proteins with vertebrates [40]. RNA interference (RNAi) was the most commonly used approach to address gene function; however, tissue-specific knockdowns have recently been developed [41]. Another reason for its wide usage as a model system is that simple phenotypes can be observed, easing the analysis of most genetic screens.

Many key factors that are capable of regulating longevity have been discovered using this model organism [42,43]. In 1993, it was demonstrated that mutations at the daf-2 locus, which encodes an ortholog of the insulin/IGF1 receptor, doubles the life span of the animal [44,45,46]. Further molecular characterization revealed that the life-span extension observed with defective daf-2 alleles required the activity of the daf-16 gene product [44]. The latter is a transcription factor of the FOXO (forkhead box transcription factor O) family, giving the first example of transcriptionally regulated aging modulation [47]. The FOXO family is an evolutionary conserved group of transcription factors that target the protein kinase Akt. In the presence of growth stimuli, FOXO proteins are first exported from the nucleus to the cytoplasm, and then degraded via the ubiquitin–proteasome pathway. On the contrary, in the absence of growth stimuli, FOXO proteins are imported to the nucleus, where they up-regulate a group of target genes, ultimately promoting cell cycle arrest, stress resistance, or apoptosis. Stress stimuli also trigger the relocalization of FOXO factors into the nucleus, thus allowing an adaptive response to stress stimuli. Additional studies identified the PI-3 kinase signaling pathway as the downstream molecular cascade target of the DAF-2 receptor, provoking the nuclear localization of the mentioned DAF-16 transcription factor [48,49,50]. HSF1, the *C. elegans* orthologue of the heat shock transcription factor, plays a critical role in the DAF-2 observed regulation of longevity. It has also been demonstrated that HSF-1 is capable of increasing the life span of DAF-2 pathway mutants, suggesting that heat shock proteins may have a pleiotropic role on pathways other than the daf-2 molecular cascade [51]. At the same time, mutations in daf-18, which is a homolog of PTEN (Phosphatase and tensin homolog) phosphatase that normally negatively regulates the insulin-dependent signal, suppress the increased survival due to mutations on DAF-2 and age-1 loci [52,53]. The latter codes the *C. elegans* catalytic subunit of the phosphatidylinositol-3-OH kinase (PI(3)K), whose impairment triggers constitutive Dauer status (the larva goes into a type of stasis and can survive harsh conditions), and increases life span and stress resistance. The lengthening of the survival of these mutants has been related to their increased resistance to oxidative stress; in fact, DAF-2 mutants express high levels of antioxidant enzymes such as catalase and superoxide dismutase, as well as low levels of free radicals. Mutants at the age-1 locus prevent the age-related decrease of catalase levels [54]. Other proteins that have been identified as important for longevity in nematode include the protein kinase phosphatidylinositol-dependent 1 (PDK1), the kinase Akt, and the mitochondrial enzymes Clk involved in ubichinone synthesis. In particular, the reduction in Clk-1 function produces smaller worms that live 15–30% longer than wild types, which is probably thanks to a decrease in basal metabolism and oxidative damage [55].

## 4. *Drosophila melanogaster*

*Drosophila melanogaster* has a 100 year-long history as an important model organism for studies on genetics and molecular biology. In 1908, Thomas Hunt Morgan was the first to use *Drosophila* as a model organism, demonstrating that genes are located on chromosomes. The fruit fly has also been the first organism for which a genetic map was obtained, thanks to Alfred Sturtevant and Calvin B. Bridges. *Drosophila melanogaster* has a generation time of 10–14 days and shows a high rate of reproduction. Large numbers of flies can easily be cultivated. Maintenance is quite simple and cost-effective. *Drosophila*’s genome is very simple: four pairs of chromosomes, 13,000 genes, and about 170 Mbp; it is about 20-fold smaller than a typical mammalian genome, but in spite of that, it encodes approximately the same number of gene families, thus making it easier to study gene function. Furthermore, many pathways, tissues, and organ systems in *Drosophila* are shared by mammals; approximately 60% of the genes that are known to be involved in human diseases have functional orthologues in the fruitfly. Thus, *Drosophila* represents one of the most useful and popular model organisms for research on human diseases. Aging research further benefits from its short life span (4–6 weeks), which allows manipulating and observing several generations while monitoring the effect of drugs, nutrients, genetic manipulation, as well as environmental factors over time. Other advantages are the wide range of tools to modulate gene function (such as mutagenesis screens, RNA interference, and transgene expression in specific stages of life or in selected tissues) and resources (cell lines, clone libraries, antibodies, microarrays, and databases) [56,57].

Studies on *Drosophila* show that longevity increases as a result of the over-expression of genes involved in stress response such as hsp70 (which encodes the heat shock protein 70), MnSOD (producing the superoxide dismutase), and mei-41 (involved in DNA repair) [58]; meanwhile, the loss of function of the pathway that includes the hormone receptor Dts3 and the insulin receptor (InR), Chico (which is the substrate of InR [59,60]), and the transcription factor dFOXO, greatly increases the life span [61]. Giannakou and Hwangbo in 2004 demonstrated that the tissue-specific overexpression of FOXO in gut and fat extends the life span, which is likely through the transcriptional repressor aop [62], which shares some targets with FOXO. Among these, Obp99b seems to have a key role, since it is upregulated in a long-lived *Drosophila* model [63]. mTOR inhibition also by rapamycin administration [64] extends life spans [65], but only in the presence of the s6 kinase [65,66]. The life extension by rapamycin treatment also requires an intact autophagy pathway [64,67]. Dietary restriction can increase the longevity of *D. melanogaster* by up to 30% as well as reduce the reproduction rate [68]. The restricted intake of specific nutrients, particularly proteins, may mediate the benefits as well as alter the macronutrients ratio. Amino acids and some amino acids sensors, such as Gcn2, seem to have a key role in life-span extension by dietary restriction [69].

## 5. Mouse

Domestic mouse is the most widely used mammalian model. Its genome is almost the same size as the human one (2.5 Gbp and 40 chromosomes), and encodes essentially the same number of genes. Most (85%) of protein-coding regions of the mouse genome are identical to human genome; in addition, 99% of the 25,000 genes have a human orthologue. Compared to other model organisms, working with mice is more difficult in every respect. They are bigger, have a generation time of about 8–10 weeks, and produce, on average, only six to eight young per brood. These numbers are interesting when compared to other mammals, but aren’t when compared to simpler model organisms. Colonies of mice are expensive to maintain, and their genetic manipulation is quite difficult. However, unlike the fruit flies and nematodes, mice have an immune system, musculoskeletal apparatus, endocrine system, digestive system, and even nervous system similar to humans both in function and architecture. A fundamental tool to explore the genetics of aging in mammals is the identification of single-gene mutations that increase life span. The first of these was discovered in 1996 studying the Ames dwarf mouse [70]. Ames dwarf mice produce a reduced amount of growth hormone (GH), prolactin, and thyroid-stimulating hormone; they have a recessive point mutation in the Prop1 gene leading to hereditary dwarfism [71]. Prop1 encodes a protein required for the pituitary activation of Pou1f1, which is a member of the POU family of transcription factors implicated in mammalian development [72]. In 1996, Brown-Borg et al., following the life span of 62 Ames dwarf mice, showed a mean life span increased of 49% and 68% in males and females respectively, and a maximal life span extension of 20% and 50%, respectively [70]. This was the first demonstration that a single gene mutation can extend the life span in mammals. The Snell dwarf mouse was described in 1929; it has a recessive spontaneous point mutation in the Pou1f1 gene that causes hereditary dwarfism [73]. Flurkey et al. in 2001 followed the life span of 24 Snell dwarf mice together with 33 normal controls. He discovered that the mean life span was extended in the dwarf group by 48%, and some of the common age-related declines were delayed [74,75]. Other studies highlight that the dwarf mice have alterations in insulin/IGF-1 signaling: both Ames and Snell dwarf mice have severely reduced circulating levels of insulin, IGF-1, and glucose [76,77]. In addition, the dwarf mice show a slower metabolism [78]. Most of the dwarf mice are also infertile [79], but they display a lower spontaneous mutation frequency [80] and a very low incidence of malignant lesions compared to controls at the time of death [81]. Notably, dwarf mice have reduced levels of DNA and protein oxidation in the liver compared to control mice [82], according to increased levels or activity of catalase and Cu/Zn superoxide dismutase in various tissues at different ages [83,84], which makes these mice resistant also to the effects of chemical stressors [85], and shows low levels of reactive oxygen species. However, the importance of oxygen concentration as a driver of protein and DNA damages leading to cellular senescence has recently been challenged [86]. In fact, it has been demonstrated that while lab mice-derived primary fibroblasts are sensitive to oxygen concentration, primary fibroblasts from wild-caught mice are not. In addition, cells from other wild-caught rodents have lower oxygen sensitivity than lab mice-derived cells [86]. This observation underlines the need to use multiple model systems and the necessity, when possible, to confirm the results on wild-caught animals, since the repeated breeding and the laboratory condition of lab animals may trigger unwanted genetic drift.

An example of the results confirmed in multiple systems are the metabolic pathways involved in the aging of *Drosophila* and *C. elegans*, which were confirmed in mice thanks to genetic manipulation. Among the genes whose deletion significantly increases the survival of mice, some of the genes involved in stress resistance have been identified, such as for example GPx4, which codes for glutathione peroxidase, and some that are part of the insulin pathway, such as FIRKO (insulin receptor in adipose tissue) [87]. Animals that have mutations in the growth hormone receptor also live longer and show a lower incidence of age-related cognitive impairment and improved insulin sensitivity [88], while those who over-express GH show signs of accelerated aging [77]. Also, female Irs-1 knock-out mice (Irs-1 is the major intracellular effector of insulin) live 32% longer than the wild-type counterpart. Finally, inhibition of the mTOR (mammalian target of rapamycin) pathway, which was obtained by rapamycin administration through S6 kinase deletion, increases survival and reduces the incidence of age-related diseases, including immune dysfunction and insulin resistance [89]. Inactivation of the PKA pathway also increases survival and causes a reduction of tumor incidence and insulin resistance over time. Another key aging gene in mice is klotho. This protein is a circulating factor that inhibits the intracellular insulin/IGF-1 signaling cascade, but its function is still controversial. Homozygous klotho-deficient mice display a syndrome similar to human progeria and anomalies in different tissues. These mice are short-lived and infertile; they show growth retardation, premature thyme involution, ectopic calcification, skin atrophy, arteriosclerosis, osteoporosis, and pulmonary emphysema [90]. On the other hand, klotho gene over-expression extends mice life span by 20–30% [91]. Higher levels of klotho are associated with longer life span, reduced atherosclerosis risk, and better hearing than other mouse strains [92]. Some mutants with mutations in DNA maintenance, stability, and repair exhibit premature aging phenotypes. Wrn knock-out mice lightly display characteristics of premature aging, including the contemporaneous deletion of the Terc gene, which encodes the RNA component of the telomerase enzyme, and brings Werner’s phenotype [93].

## 6. Domestic Dog

The domestic dog is a very interesting aging model for different reasons. Dogs allow us to observe them in their natural environment while being investigated; notably, they often share lifestyle and sometimes exercise habits with humans. Canine life span can vary greatly [94]. Interestingly, it has been observed that life span inversely correlates with body size. Larger breeds live almost six to seven years, while smaller breeds can reach up to 16 years [95,96,97]. Dogs share the same diseases with humans, in particular age-related diseases such as congestive heart failure, renal and hepatic disease, sarcopenia, diabetes, obesity, joint disease, neurodegeneration, cataracts, immune-mediated illnesses, and cancer [97,98,99,100,101]. In addition, it is the only species where massive breed selection has led to large numbers of individuals with very small phenotypic and genotypic differences within a specific breed, but with very large differences between different breeds. Maybe as a result of this strong selection for specific traits, some pure breeds of dogs are more prone to specific age-related diseases than others [97]. For example, the analysis of cancer incidence in different pure breeds confirmed that larger breeds have a higher incidence of cancer but revealed also that each breed has increased incidence for specific cancer types, suggesting that anatomic differences and genomic differences between breeds can explain these differences. It is also interesting to know that as a companion animal, dogs often share part of their lifestyle with their owners, including physical exercise. At the same time, dogs receive medical treatments from veterinarian specialists during their life as it happens for humans, and that contributes to increase the life span of these companion animals. The domestic dog has a well-annotated genome that was fully sequenced 10 years ago [102,103,104]. The size of the diploid genome is 4900 Mbp, which is organized in 38 pairs of autosomes and two sex chromosomes. Different genetic resources are applied in canine genetic research. Furthermore, genetic pedigrees are registered for many generations [105]. Greer et al. (2007) reported a correlation between body size and longevity. They noticed that smaller members (Chihuahua) live longer than larger members (e.g., Great Dane) [95]. Similar data have been obtained from studies in a murine system in which the IGF/GH axis resulted involved in longevity and body size determination [106]. Notably, circulating IGF-1 levels correlate with the body size of adult dogs; in fact, IGF-1 levels increase with body weight. Therefore, the data is consistent with previous work by Eigenmann et al. (1988), indicating that adult dogs have a high correlation between circulating IGF-1 and adult body size [107]. Furthermore, IGF-1 decreases over time as a function of age [108]. In addition, a reduction of the insulin/insulin-like growth factor-1 (IGF-1) signaling cascade significantly extends life span [109]. Interestingly, Greer et al. described that serum IGF-1 levels decrease at a higher rate in intact females than in spayed females. Similarly, there is a significant difference in the serum IGF-1 levels between neutered and intact males. For both intact males and females, an increase in overall body weight was significantly associated with higher levels of IGF-1. These data suggest relevant hormonal effects on IGF-1 action [104]. Interestingly, Waters et al. (2009) demonstrated that dogs with four years or more of ovarian hormone exposure live longer than ovariectomized dogs [110]. Therefore, higher body weight is related to high level of serum circulating IGF-1, which in turn seems to be deleterious for aging. Accordingly, the maintenance of lean body mass and reduced accumulation of body fat have been associated with attaining a longer than average life span [111]; thus, dog’s weight may be more predictive of life span than height, as reported by Greer et al. [95]. A dietary restriction of 25% has been reported to increase life span by about two years [112,113], improving metabolic health [114,115] and delaying immune senescence in Labrador retrievers maintained in a laboratory environment [114,115]. Further studies will be necessary to discriminate as to whether increasing the health and life span after CR in dogs is the effect of weight loss or depends on the down-regulation of pro-aging pathways such as previously demonstrated in simple model organisms such as yeast. Actually, studies carried out by Jimenez et al. suggest that large breed dogs may have higher glycolytic rates, and an increased DNA mutation rate, which could be responsible for their decreased life span compared with small breed dogs, despite reactive oxygen species (ROS) production showing no differences across size and age classes [116]. Likewise, Alexander et al. observed a decline in the heat shock protein 70 response after heat stress with age, suggesting a role of oxidative stress in dog’s aging [117].

## 7. Non-Human Primates

The models closest to humans are non-human primates. In fact, the chimpanzee genome has more than 98% homology with the human genome, while the rhesus monkey (*Macaca mulatta*) has 93% DNA homology with the human genome [118,119]. The Macaca mulatta genome is 3146 Mbp and codes 21,000 different proteins [120]; *Pan troglodytes*, a common chimpanzee, has a 3385-Mbp genome coding for 23,534 proteins. Interestingly, they have a similar inter-individual variability to humans and share eating and sleeping behavior, physiology, neurology, endocrinology, immunology, as well as anatomy [121]. Their average life span ranges from 7 to 30 years depending on the species considered, while the maximum life span of the commonly used macaque reaches 40 years, and the chimpanzees can reach up to 65 years [122]. Similarly to humans, monkeys exhibit signs of physical decline and many age-associated diseases, including [123]: sarcopenia [124], osteoporosis [125], cataracts, cardiovascular diseases, and cancer [123]. In fact, the incidence of these diseases is similar to that in humans. Researchers take advantage of two important resources: the Internet Primate Aging Database, which collects data such as body weight and blood chemistry measurements on many non-human primate species during aging, and the Nonhuman Primate Tissue Bank. Another advantage is the possibility to fully control the environment, dietary intake, and medical history. On the other hand, non-human primates require specialized care, have high cost, and require ethical considerations. However, they represent the ideal model to summarize the complex in vivo physiology of human aging. Even though research such as direct manipulation of the monkey genome are not possible for ethical reasons, observational studies and dietary interventions may give us very useful information, especially to validate in the closest to human system what has been previously discovered in simpler organisms. To this purpose, three major studies have been performed in non-human primates to evaluate the efficacy and safety of calorie restriction and aging: one was performed at the University of Maryland [126], one was at NIA (National institute of Aging) [127], and the last one was at the Wisconsin National Primate Research Center [128]. All of them confirmed the benefits of CR on health. The University of Wisconsin study confirmed that CR reduces disease incidence and prolongs life span [129,130,131], while the NIA study doesn’t observe a different survival between CR and control monkeys. Differences in feeding protocol, diet composition, and the timing of CR onset may explain the observed differences [132]. However, data from both studies suggest that many of the beneficial effects of CR reported in rodents also occur in primate models, thus suggesting a possible role also in human aging [131].

From comparative genetics studies between non-human primates and humans, it is deducible that some of the genetic regulatory processes that are important during development are less subjected to selective pressure and may became adverse later in life [133]. Blalock et al. analyzed gene expression in two major hippocampal subdivisions that are critical for memory/cognitive function in rhesus monkeys, identifying genes that changed expression with aging, and showed that increased gene expression of the glucocorticoid receptor happens with aging in rhesus macaques, linking the age-dependent metabolic syndrome to aging changes in the brain [134]. Epigenetic changes occur during aging in monkeys. In rhesus macaque brains, aging is associated with a global increase in H3K4me2 and H3K4me3 transcriptional activation; in addition, SETD7 and DPY30 (H3K4me2 methyltransferases) show elevated expression [135]. A role in aging has been ascribed to miRNA; in particular, Mohan et al. identified the miR-34a-SIRT1-acetylp65 axis as a potential mediator of “inflammaging” in the intestine [136].

## 8. Discussion

Humans are not an easy-to-study system both for ethical as well as for practical reasons, and have required the support of simpler organisms to model their physiology and pathology. Epidemiology, one of the most used approaches to investigate humans, is the branch of science that tries to count how often a certain pathology occurs in different population groups, hopefully identifying the risk or protective factors against certain pathologies. It is an essential methodology to study humans, but can fall short because it often requires a very large cohort of people, is normally very time and budget consuming, and can potentially be affected by many confounding factors. Clinical trials often use small groups of people to be cost effective and test specific hypotheses ranging from the safety/effectiveness of selected drugs to specific devices. Of course, obvious ethical as well as practical issues limit the potential of this approach in humans. Therefore, although both approaches are essential for the understanding of human physiology and pathology, they unlikely may suggest alone new branches of science or help to develop breakthrough ideas [137]. On the other hand, human genetics has been very successful, and revealed the genetic cause of more than 6000 monogenic diseases in humans, but it is hard to imagine the development of this science without the seminal discoveries in plants, fruitfly, fungi, etc., also in humans. The identification of genes and pathways relevant for human longevity has acquired deep advantages from model systems and discoveries, which can be grouped in four functional categories:

- Genes related to stress resistance: their role in longevity has first been demonstrated in many different model systems [18,24,25,32,33,34,51,54,58,82,83,84,85] and eventually confirmed in centenarians who show a low degree of oxidative stress as well as high antioxidant protection [138,139]. A high level of oxidative stress is also an important risk factor of other age-related diseases such as hypertension, atherosclerosis, and diabetes. SNP (single nucleotide polymorphisms) studies have identified Tp53, coding for tumor suppressor p53 [140,141,142], EXO1 [143], GPX1 (glutathione peroxidase1) [144], SOD2 (manganese superoxide dismutase) [145], heat shock proteins genes HSPA1A, HSPA1B, and HSPA1L [146,147,148], GSTZ1 (glutathione S-transferase zeta 1) [149], NOS1, NOS2 (nitric oxide synthase 1 and 2) [150], and UCPs (uncoupling proteins) [147,151,152] as susceptibility genes.
- Genes involved in telomeres length: they have been found to be associated with human longevity such as TERT and TERC (telomerase reverse transcriptase, telomerase RNA component) [153], SIRT1, and SIRT3 (sirtuins) [154,155]. The first discoveries were made in yeasts and tetrahymena by Elizabeth Blackburn, finding the role of TERT and TERC ([156] and references within). In *Caenorhabditis elegans* over-expressing a protein involved in telomere length regulation leads to the elongation of telomeres and extends the life span, making the organism more resistant to heat stress [157]. The over-expression of TERT also extends the life span of mice [158]. In yeast, sirtuins promote longevity [159]; in particular, it has been reported that Sir2 mediates life-span extension due by calorie restriction [160]. These findings have been replicated in other model organisms [161], but their role in longevity is not consistent for all species, and therefore is still under debate [162].
- Genes involved in metabolism and cellular division: APOE (apolipoprotein E) [163], TXNRD1 (thioredoxin reductase 1), XDH (xanthine dehydrogenase) [163], MAP3K7 (mitogen-activated protein kinase kinase kinase 7) [149], AKT kinase, and TOR [164]. The association of APOE with human longevity have been replicated in different populations: [165,166,167]. Apolipoprotein E (apoE) exhibits three isoforms: apoE2, apoE3 and apoE4. They are involved in inflammation, elevated lipid levels, and oxidative stress; furthermore, these are risk factors for cardiovascular disease and Alzheimer’s disease, as reported by Huebbe et al. (2011) [168]. APOE2 has been defined as a longevity gene for its putative protective function; it is abundant in long-lived people, while APOE4, that differs from e3 allele at a single aa (112cys), and has been considered a frailty allele [169]. In fact, it increases the risk of Alzheimer’s disease and cardiovascular diseases, maybe for a putative interaction with the *β amyloid* protein, and it is almost absent in centenarians.
- Genes belonging to the IGF/GH and insulin pathway: mutations in genes belonging to the insulin or insulin-like signaling pathway extend the life span of *Caenorhabditis elegans* [170,171], *Drosophila melanogaster* [59,109,172], and mice [69,173]. In humans, it has been observed that insulin sensitivity normally decreases during aging. On the other hand, centenarians are more sensitive to insulin than other people, and often show lower IGF-1 plasma levels [174]. SNP studies have found an association of particular alleles or haplotypes for INS (insulin) [175], INSR (insulin receptor) [176], IGF1 (insulin growth factor 1) [177], IGF1R (insulin growth factor 1 receptor); in fact, a specific haplotype of the IGF-I receptor and the kinase PI3KCB is frequently found in individuals living longer together with low plasma levels of IGF-1 [178], IGF2 (insulin growth factor 2) [179], IGF2R (insulin growth factor 2 receptor) [180], IRS1 (insulin receptor substrate 1) [177], GH1 (growth hormone 1) [177], GHSR (growth hormone secretagogue receptor type 1) [175], FOXO1A (forkhead box protein O1 A), and FOXO3A (forkhead box protein O3 A) transcription factor, which contains alleles that are associated with longevity in multiple Asian and European populations [181,182,183,184,185].

FOXO is a transcription factor that is conserved in all eukaryotic organisms and is negatively regulated by the insulin-signaling pathway. When insulin or insulin-like growth factor signaling is low, FOXO is activated, and life-span extension occurs [182].

Studies on all of the model organisms cited above have thus contributed to the discovery of the fundamental mechanisms of aging in humans. The essential conservation of these mechanisms throughout evolution has been strikingly confirmed in all the model organisms that have been tested so far. The series of similarities found in the mechanisms of regulation of aging in all the model organisms and in humans make us believe that these mechanisms have been preserved during the evolution from yeast to mammals [36,186]. For example, the protein sequence of DAF-2 (the *C. elegans* insulin/IGF receptor ortholog) shows 34% identity with the IGF-IR of mammals, nematode’s AGE-1 is 27% identical to its ortholog PI3KCB kinase, and DAF-16 is 49% identical to FOXO1A, while IRS-1 has 30% identity with *Drosophila*’s insulin receptor substrate 1 (CHICO). In addition, these factors regulate similar processes in all organisms such as resistance to oxidative stress, metabolism, nutrient utilization, and of course life span [187]. A 2007 study compared genes whose transcription varies with the inhibition of the insulin/IGF-1 pathway in three different species—*C. elegans*, *Drosophila*, and mouse—and it was demonstrated that there are significant similarities concerning in particular two main categories of genes. The first one includes genes involved in protein synthesis that are hypoexpressed (this was also independently observed in yeast [188]), the second one includes genes involved in detoxification that are over-expressed, (e.g., the gene coding for glutathione S-transferase [189]). Consistent with the latter observation, the over-expression of transcription factors that regulate xenobiotics metabolism increases survival in *Caenorhabditis* and *Drosophila* [190]. The considerable affinities that were found confirm that the mechanism of aging, which is specifically mediated by the insulin-dependent pathway, has been preserved during the evolution in all eukaryotes from yeast to humans. In addition, it has been also confirmed that these pathways may be regulated mainly by nutrients [191]. Calorie restriction, protein restriction, fasting, and a fasting-mimicking diet are in fact becoming interesting alternatives to manipulating our genome structure [192]. Again, it has been so far confirmed that simple model systems, despite their clear morphological differences with humans, can be effectively used as a model to pave the way to future relevant discoveries in humans. Therefore, we believe that model systems will continue to be an essential tool for aging research and for the usage of high-throughput methodologies However, the observation that wild-caught animals may behave differently than lab animals stresses the need to confirm the results obtained with one organism in other species or in wild-caught animals in order to avoid the possibility that lab condition and repeated breeding may have favored genetic drift or epigenetic changes.
